# Impaired Treg function and activation of the CXCL10/CXCR3 inflammatory pathway in Non‐Fluent Variant Primary Progressive Aphasia

**DOI:** 10.1002/alz70855_101037

**Published:** 2025-12-23

**Authors:** Nazaret Gamez, Abdulmunaim M Eid, Belen Pascual, Joseph C. Masdeu, Stanley H Appel, Alireza Faridar

**Affiliations:** ^1^ Houston Methodist Research Institute, Houston, TX, USA

## Abstract

**Background:**

Despite growing evidence supporting the role of inflammation in neurodegenerative processes, a detailed molecular understanding of the peripheral immune system's status and its mechanistic implications in the pathology of frontotemporal dementia (FTD) remains limited. In this study, we investigate (a) the suppressive function of regulatory T cells (Tregs), (b) the inflammatory profile of peripheral monocytes, and (c) the levels of inflammatory markers in the plasma of individuals with non‐fluent variant primary progressive aphasia (nfvPPA), a subtype of FTD.

**Method:**

Plasma and peripheral blood mononuclear cells (PBMCs) were obtained from 17 nfvPPA and 34 age‐matched HC recruited by the Houston Methodist Nantz National Alzheimer's Center. The suppressive function of Tregs was assessed using proliferation assays. RNA from peripheral monocytes was analyzed via Nanostring chips and qRT‐PCR to determine the inflammatory profiles. Plasma cytokine levels were measured using the Olink® Target 48 Cytokine panel. Time‐of‐flight mass cytometry (CyTOF) was employed to evaluate PBMCs immunoprofiles and the activation of signaling pathways at single‐cell resolution.

**Result:**

Tregs from nfvPPA individuals demonstrated a compromised ability to suppress responder T cell proliferation compared to HC. Additionally, peripheral monocytes in the nfvPPA cohort exhibited significantly increased expression of pro‐inflammatory genes, including *C1q, NLRP3, PRG3*, and *CXCL10*. Elevated levels of *CXCL10* were also detected in the plasma of nfvPPA patients. By integrating both transcriptomic and proteomic data, we identified the activation of the CXCL10/CXCR3 cascade in nfvPPA individuals. Proteomic analysis by CyTOF revealed that CXCL10 was predominantly expressed by CD14 monocytes, while its receptor, CXCR3, was highly expressed on CD4 and CD8T cells. Our i*n vitro* studies demonstrated that CXCL10 exerted pro‐inflammatory effects on CXCR3‐positive T cells, effects that were mitigated by the addition of the selective CXCR3 blocker AMG487.

**Conclusion:**

Individuals with nfvPPA exhibit impaired immunosuppressive function of Tregs, accompanied by systemic activation of the CXCL10/CXCR3 pathway, which is involved in the homing of T cells into the brain. Our findings highlight the potential clinical impact of (i) restoring Treg suppressive function and (ii) targeting the CXCL10/CXCR3 axis to mitigate neurodegeneration by limiting neuroinflammation and the infiltration of cytotoxic T cells to the brain.

